# Construction of a survival prediction model for high-and low -grade UTUC after tumor resection based on “SEER database”: a multicenter study

**DOI:** 10.1186/s12885-021-08742-3

**Published:** 2021-09-07

**Authors:** Mengmeng Wang, Xin Ren, Ge Wang, Xiaomin Sun, Shifeng Tang, Baogang Zhang, Xiaoming Xing, Wenfeng Zhang, Guojun Gao, Jing Du, Shukun Zhang, Lijuan Liu, Xia Zheng, Zhenkun Zhang, Changgang Sun

**Affiliations:** 1 Clinical Medical Colleges, Weifang Medical University, Weifang, 261000 China; 2 Department of Oncology, Weifang Traditional Chinese Hospital, Weifang, 261000 Shandong China; 3 Department of Pathology, Weifang Medical University, Weifang, 261053 China; 4 Department of Pathology, Affiliated Hospital of Qingdao University, Qingdao, 266555 China; 5 Department of Urology, The Affiliated Hospital of Weifang Medical College, Weifang, 261000 China; 6 Department of Urology, Weifang Traditional Chinese Hospital, Weifang, 261000 China; 7 Department of Pathology, Shandong University,Weihai Municipal Hospital, Cheeloo College of Medicine, Weihai, 264200 China; 8 Department of Oncology, ShouGuang People’s Hospital, Weifang, 262700 China; 9 Qingdao Academy of Chinese Medical Sciences, Shandong University of Traditional Chinese Medicine, Qingdao, 266112 Shandong People’s Republic of China

**Keywords:** Upper tract urothelial carcinoma, SEER program, Grade, Nomogram, Tumor resection, Overall survival, Validation, Multicenter

## Abstract

**Background:**

There are differences in survival between high-and low-grade Upper Tract Urothelial Carcinoma (UTUC). Our study aimed to develop a nomogram to predict overall survival (OS) of patients with high- and low-grade UTUC after tumor resection, and to explore the difference between high- and low-grade patients.

**Methods:**

Patients confirmed to have UTUC between 2004 and 2015 were selected from the Surveillance, Epidemiology and End Results (SEER) database. The UTUCs were identified and classified as high- and low-grade, and 1-, 3- and 5-year nomograms were established. The nomogram was then validated using the Chinese multicenter dataset (patients diagnosed in Shandong, China between January 2010 and October 2020).

****Resu**lts:**

In the high-grade UTUC patients, nine important factors related to survival after tumor resection were identified to construct nomogram. The C index of training dataset was 0.740 (95% confidence interval [CI]: 0.727–0.754), showing good calibration. The C index of internal validation dataset was 0.729(95% CI:0.707–0.750). On the other hand, Two independent predictors were identified to construct nomogram of low-grade UTUC. The C index was 0.714 (95% CI: 0.671–0.758) for the training set,0.731(95% CI:0.670–0.791) for the internal validation dataset. Encouragingly, the nomogram was clinically useful and had a good discriminative ability to identify patients at high risk.

**Conclusion:**

We constructed a nomogram and a corresponding risk classification system predicting the OS of patients with an initial diagnosis of high-and low-grade UTUC.

**Supplementary Information:**

The online version contains supplementary material available at 10.1186/s12885-021-08742-3.

## Introduction

Upper Tract Urothelial Carcinoma (UTUC) is a relatively rare tumor of the genitourinary system, affecting 2 in every 100,000 people [1]. It accounts for approximately 5 to 10% of urothelial malignancies [2, 3]. UTUC includes renal pelvis tumors and ureteral tumors [2]. Two-thirds of UTUC are reportedly invasive at the time of diagnosis [4]. Therefore, radical nephroureterectomy (RNU) is the gold standard for the treatment of high-risk patients with UTUC [2, 5]. However, some patients undergo minimally invasive treatment [6, 7]. Irrespective of the surgical method used, it is beneficial for the overall survival rate.

Tumor grade is a reliable predictor of cancer-related prognosis in patients with UTUC because it is closely related to cancer aggressiveness and tumor stage. At the same time, it is also an important predictor of postoperative survival [8, 9]. According to the World Health Organization (2004/2016) classification, UTUC is divided into high- and low-grade [2], which is different from the World Health Organization (1973) classification that included G1, G2, and G3. The high- and low-grade tumor grading system is a strong independent predictor of UTUC recurrence and death. In a previous analysis, high-grade tumors were found to be more aggressive than the low-grade tumors. Patients with high aggressive tumors have a lower survival rate [10], whereas those with low aggressive tumors have a higher survival rate.

The rationale for conducting postoperative evaluation is that interventions can be directed toward those patients most likely to benefit. For example, more thorough therapeutic interventions can be implemented for patients with high-grade tumors, as well as prevention and early treatment for patients with low-grade tumors. Several groups have previously published nomograms that predict the outcome of tumor resection in patients with UTUC [11, 12]. However, these studies treated high-grade and low-grade as a single group and did not discuss the differences in survival prediction models between the two grades. Although some studies have carried out survival model prediction for high-grade patients, there is still a lack of low-grade models. Therefore, for clinical application, it is not easy and direct to conduct real-time and accurate evaluation for patients of different grades, leading to limitations in the clinical applicability of UTUC prediction models. In addition, there is still a lack of understanding of the overall survival characteristics of patients with low- and high-grade tumors, and clinical trials are needed to conduct postoperative evaluation and validation.

Due to the rarity of UTUC, trends in disease incidence, associated demographic factors, and predictors of cancer-related and overall survival outcomes are limited. Although a previous study reported differences in survival and clinical characteristics between patients with high- and low-grade tumors, it was a single center study with a small sample size that was not representative [13]. Based on these considerations, we relied on the Surveillance Epidemiology and End Results (SEER) database of patients classified as high-grade and low-grade after tumor resection to build predictive models. More importantly, we validated this model using the Chinese multicenter external verification dataset.

## Methods

### Patient selection

**Fig. 1:**
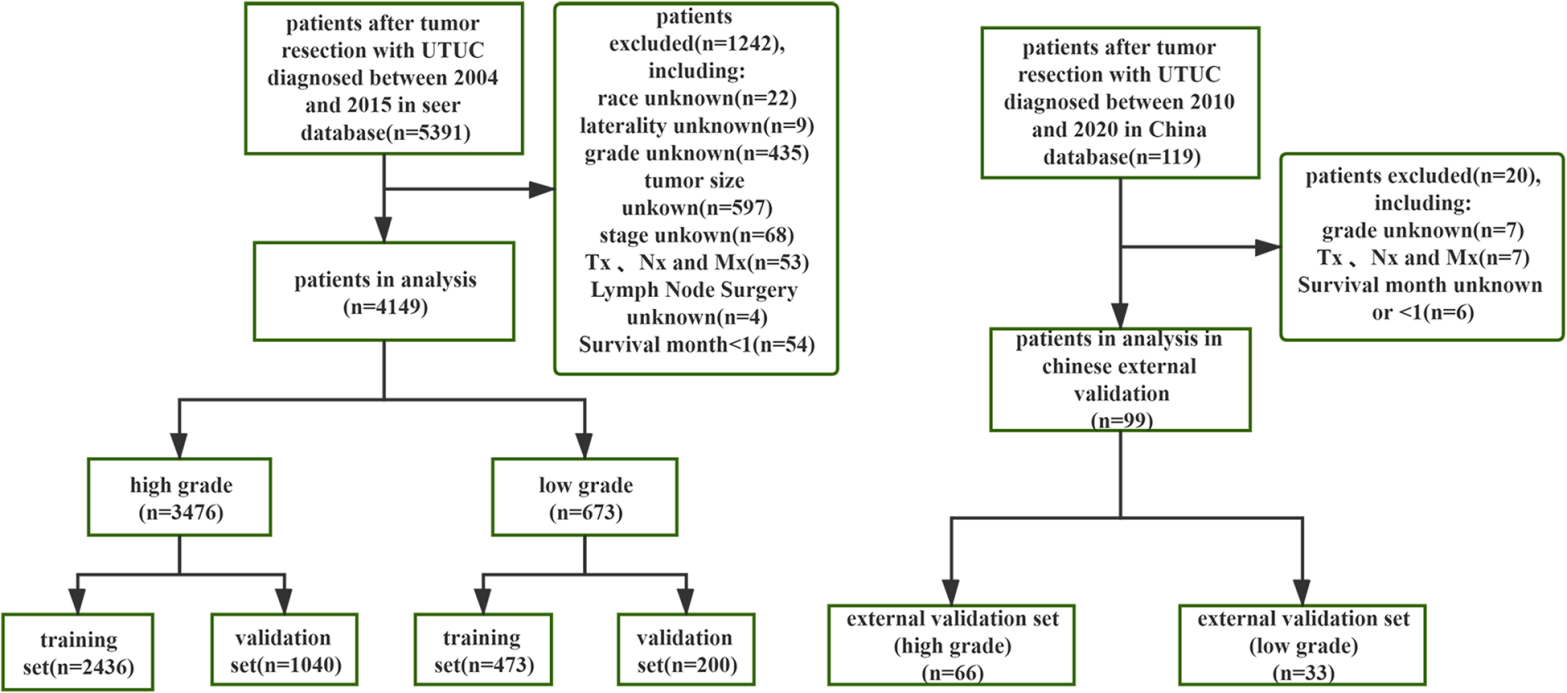
Flow chart illustrating patient selection for this study

In this multicenter retrospective study, we retrieved data for patients with UCTC from the SEER database. The case recruitment method is shown in Fig. 1. The criteria for data extraction from the database were: (1) patients diagnosed with UTUC between 2004 and 2015; (2) the only or the first primary tumor confirmed by histology was UTUC,no history of bladder cancer or radical cystectomy;(3) SEER records for which the International Classification of Diseases for Oncology, third edition (ICD-O-3) codes included:“C65.9-Renal pelvis”,“C66.9-Ureter”;(4)(ICD-O-3) morphology:8020/3, 8031/38082/38120/38122/38130/38131/3;(5) tumor resection was performed; (6) clinicopathological and follow-up data were available. The exclusion criteria included: (1) patients with missing or incomplete data, such as tumor grade, survival status and survival time, sex, laterality, race, American Joint Committee on Cancer (AJCC) stage, T stage, N stage, M stage, primary site and Lymph node dissection;(2) patients followed up at 1 month or less than 1 month after initial diagnosis. Finally, eligible patients with UCTC in the SEER database were identified and classified as high or low-grade.

In addition, a Chinese multicenter verification dataset (cancers diagnosed in three hospitals affiliated to Weifang Medical College, Weifang traditional Chinese Medicine Hospital,Weihai Municipal Hospital and People’s Hospital Of Rizhao between January 2010 and October 2020) was used for external verification. Through the hospital electronic medical record system, we have collected basic patient data, clinical and pathological information, and survival status. Except for the year of diagnosis, the inclusion and exclusion criteria of the external validation dataset were the same for the Chinese multicenter dataset. The information is anonymous and has been approved by the ethics Committee of Weifang Hospital of Traditional Chinese Medicine.

### Data collection

Demographic and clinical data were retrieved, including age, sex, race, grade, primary site of the tumor, laterality, tumor size, AJCC TNM stage, AJCC stage, histology type, Lymph node dissection, radiotherapy, and chemotherapy. The AJCC and TNM stages were reclassified according to the AJCC 8th edition staging criteria [14]. We used X-tile software to determine the best cut-off value for age at diagnosis and tumor size.

The aim of UTUC treatment is to improve overall survival (OS) and achieve eventual cure. Therefore, the clinical endpoint of this study was OS, defined as the time from surgery to death, or the date of last follow-up for those patients who were alive and reviewed.

### Development, performance assessment and validation of the nomogram

In the training set, univariate Cox regression analysis was used to test the predictors and multivariate Cox regression analysis was used to construct the pre-modeling of high-grade and low-grade OS respectively. Model 1 included AJCC and TNM stages, and model 2 included AJCC stage. The stopping rule of the backward stepwise selection is guided by Akaike’s Information Criterion (AIC) [15]. Other predictor variables were the same for the two models. We used the Harrell’s concordance index (C-index) to quantify the discrimination accuracy of the models [16]. On this basis, nomograms were established for the high-and low-grade patients. The discriminant ability of line chart was quantitatively evaluated by C-index. Calibration curves were then drawn to evaluate the calibration of the nomograms. ROC curve (time-dependent AUC) were used to evaluate the discriminant ability of the nomogram. The performance of the nomogram was verified by the SEER internal verification dataset and external dataset from Chinese multicenter database.

### Risk stratification based on the nomogram

The Kaplan-Meier method was used to compare the high- and low-grade risk stratification, risk stratification of the training dataset, and validation dataset of the nomogram, as well as analyze the risk stratification of radiotherapy. The Kaplan-Meier method was used to estimate the probability of OS occurrence.

### Statistical analysis

For comparison between groups, Univariate and multiple Cox regression analysis and OS were calculated using SPSS (IBM SPSS Statistics for Windows, Version 26.0. Armonk, NY: IBM Corp). X-tile software version 3.6.1 (Yale University, New Haven, Connecticut, USA) was used to determine the best cut-off value. Random grouping was accomplished with the “CARET” package. Survival analysis was performed by the “survival” software package. The nomogram and calibration chart are generated using the “rms” software package. R software version 4.0.3 was used for analysis (R Foundation for Statistical Computing, Vienna, Austria, https://www.r-project.org/). In a two-tailed test, statistical significance was set at *P* values less than 0.05.

## Results

### Patient characteristics

As shown in Fig. 1, the SEER database had a total of 4149 eligible records for which UTUC histologic classification was high- or low-grade. The records were randomly divided into the SEER training dataset and SEER internal validation dataset according to a 7:3 ratio. The median follow-up time for the entire dataset was 28 months (interquartile range [IQR]: 13–60.5 months). In addition, 99 eligible UTUC records were obtained from a multicenter database in China, which served as an external validation dataset for this study.

In Table 1, the median ages of the high- and low-grade cohorts were 73 and 69 years, respectively; thus, the median age of the high-grade patients was higher than that of the low-grade patients. The median tumor size (40 mm vs. 32 mm) of the high- and low-grade patients showed that the size of high-grade tumors was larger than that of low-grade tumors. Regarding the T stage, the high-grade patients were mainly in stage T3 (48.1%), whereas the low-grade patients were mainly in stage T1 (58.2%). In addition, N0 and M0 accounted for the majority in the high- and low- grade patients. In terms of treatment, 35.1% of the high-grade patients underwent Lymph node dissection, which was higher than the percentage of low-grade patients (18.4%). The proportion of patients receiving radiotherapy and chemotherapy was relatively small. Appendix Table 2 showed the percentage of surgical procedures.

**Table 1:** Patient and tumor characteristics of high-and low-grade datasets

	SEER Whole	SEER Training	SEER Validation	Chinese Validation
	Population [cases(%)]	Cohort [cases(%)]	Cohort [cases(%)]	Cohort [cases(%)]
**High grade Characteristis**				
Total	3476	2436	1040	66
**Age**				
Median (IQR),years: 73 (64–80)				
< =66	1128 (32.5)	803 (33.0)	325 (31.3)	22 (33.3)
67–80	1565 (45.0)	1097 (45.0)	468 (45.0)	36 (54.5)
> 80	783 (22.5)	536 (22.0)	247 (23.8)	8 (12.1)
**Sex**				
male	1929 (55.5)	1353 (55.5)	576 (55.4)	–
female	1547 (44.5)	1083 (44.5)	464 (44.6)	–
**Race**				
white	2979 (85.7)	2074 (85.1)	905 (87.0)	–
black	155 (4.5)	109 (4.5)	46 (4.4)	–
other	342 (9.8)	253 (10.4)	89 (8.6)	–
**Primary_site**				
Renal pelvis	2416 (69.5)	1690 (69.4)	726 (69.8)	–
Ureter	1060 (30.5)	746 (30.6)	314 (30.2)	–
**Grade**				
III	1151 (33.1)	804 (33.0)	347 (33.4)	–
IV	2325 (66.9)	1632 (67.0)	693 (66.6)	–
**Laterity**				
left	1756 (50.5)	1250 (51.3)	506 (48.7)	–
right	1720 (49.5)	1186 (48.7)	534 (51.3)	–
**Tumor_size**				
Median (IQR),mm: 40 (25–55)				
< 65 mm	2809 (80.8)	1971 (80.9)	838 (80.6)	62 (93.9)
> =65 mm	667 (19.2)	465 (19.1)	202 (19.4)	4 (6.1)
**Histology**				
Transitional	1822 (52.4)	1291 (53.0)	531 (51.1)	57 (86.4)
Papillary	1611 (46.3)	1116 (45.8)	495 (47.6)	8 (12.1)
0ther	43 (1.2)	29 (1.2)	14 (1.3)	1 (1.5)
**AJCC Stage**				
I	698 (20.1)	493 (20.2)	205 (19.7)	–
II	533 (15.3)	380 (15.6)	153 (14.7)	–
III	1286 (37.0)	886 (36.4)	400 (38.5)	–
IV	959 (27.6)	677 (27.8)	282 (27.1)	–
**T**				
T1	733 (21.1)	520 (21.3)	213 (20.5)	1 (1.5)
T2	595 (17.1)	416 (17.1)	179 (17.2)	27 (40.9)
T3	1672 (48.1)	1161 (47.7)	511 (49.1)	33 (50.0)
T4	476 (13.7)	339 (13.9)	137 (13.2)	5 (7.6)
**N**				
N0	2837 (81.6)	1982 (81.4)	855 (82.2)	53 (80.3)
N1	354 (10.2)	255 (10.5)	99 (9.5)	7 (10.6)
N2	285 (8.2)	199 (8.2)	86 (8.3)	6 (9.1)
**M**				
M0	3214 (92.5)	2251 (92.4)	963 (92.6)	61 (92.4)
M1	262 (7.5)	185 (7.6)	77 (7.4)	5 (7.6)
**Ln_surg**				
yes	1220 (35.1)	868 (35.6)	352 (33.8)	12 (18.1)
no	2256 (64.9)	1568 (64.4)	688 (66.2)	54 (81.8)
**Radiation**				
yes	246 (7.1)	165 (6.8)	81 (7.8)	4 (6.1)
no	3230 (92.9)	2271 (93.2)	959 (92.2)	62 (93.9)
**Chemotherapy**				
yes	930 (26.8)	647 (26.6)	283 (27.2)	29 (43.9)
no	2546 (73.2)	1789 (73.4)	757 (72.8)	37 (56.1)
**Low grade Characteristis**				
Total	673	473	200	33
**Age**				
Median (IQR),years: 69 (61–78)				
<=71	378 (56.2)	271 (57.3)	107 (53.5)	18 (54.5)
> 71	295 (43.8)	202 (42.7)	93 (46.5)	15 (45.5)
–	–	–	–	–
**Sex**				
male	399 (59.3)	282 (59.6)	117 (58.5)	–
female	274 (40.7)	191 (40.4)	83 (41.5)	–
**Race**				
white	591 (87.8)	419 (88.6)	172 (86.0)	–
black	33 (4.9)	21 (4.4)	12 (6.0)	–
other	49 (7.3)	33 (7.0)	16 (8.0)	–
**Primary_site**				
Renal pelvis	449 (66.7)	312 (66.0)	137 (68.5)	–
Ureter	224 (33.3)	161 (34.0)	63 (31.5)	–
**Grade**				
I	145 (21.5)	103 (21.8)	42 (21.0)	–
II	528 (78.5)	370 (78.2)	158 (79.0)	–
**Laterity**				
left	344 (51.1)	247 (52.2)	97 (48.5)	–
right	329 (48.9)	226 (47.8)	103 (51.5)	–
**Tumor_size**				
Median (IQR),mm: 32 (22.5–47.5)				
<=20 mm	147 (21.8)	90 (19.0)	57 (28.5)	–
> 20 mm	526 (78.2)	383 (81.0)	143 (71.5)	–
**Histology**				
Transitional carcinoma	160 (23.8)	117 (24.7)	43 (21.5)	–
Papillary transitional cell carcinoma	513 (76.2)	356 (75.3)	157 (78.5)	–
–	–	–	–	–
**AJCC Stage**				
I	386 (57.4)	271 (57.3)	115 (57.5)	12 (36.4)
II	112 (16.6)	79 (16.7)	33 (16.5)	9 (27.3)
III	129 (19.2)	90 (19.0)	39 (19.5)	10 (30.3)
IV	46 (6.8)	33 (7.0)	13 (6.5)	2 (6.0)
**T**				
T1	392 (58.2)	275 (58.1)	117 (58.5)	–
T2	115 (17.1)	82 (17.3)	33 (16.5)	–
T3	142 (21.1)	95 (20.1)	47 (23.5)	–
T4	24 (3.6)	21 (4.4)	3 (1.5)	–
**N**				
N0	651 (96.7)	460 (97.3)	191 (95.5)	–
N1	13 (1.9)	10 (2.1)	3 (1.5)	–
N2	9 (1.3)	3 (0.6)	6 (3.0)	–
**M**				
M0	659 (97.9)	464 (98.1)	195 (97.5)	–
M1	14 (2.1)	9 (1.9)	5 (2.5)	–
**Ln_surg**				
yes	124 (18.4)	79 (16.7)	45 (22.5)	–
no	549 (81.6)	394 (83.3)	155 (77.5)	–
**Radiation**				
yes	9 (1.3)	8 (1.7)	1 (0.5)	–
no	664 (98.7)	465 (98.3)	199 (99.5)	–
**Chemotherapy**				
yes	57 (8.5)	44 (9.3)	13 (6.5)	–
no	616 (91.5)	429 (90.7)	187 (93.5)	–

Data are n or n (%) unless indicated otherwise. *AJCC* the American Joint Committee on Cancer, *Ln_ dissection* Lymph node dissection

### Development and performance evaluation of nomogram

**Fig. 2:**
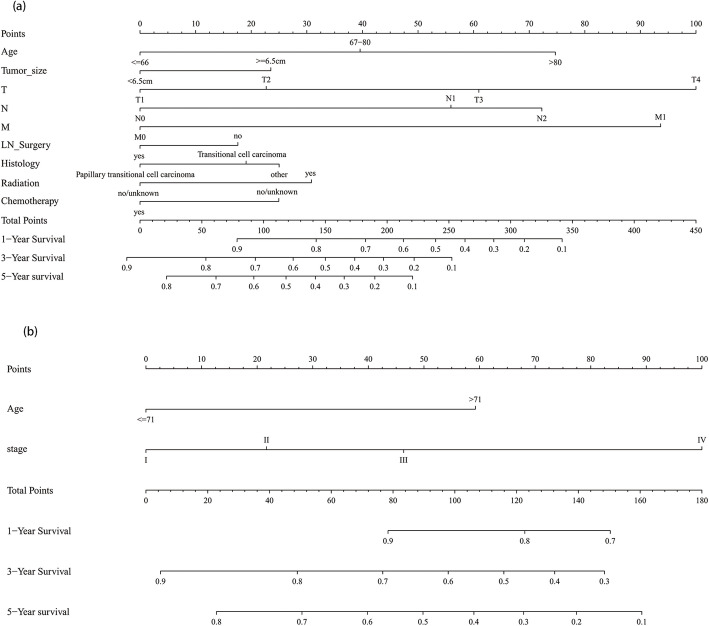
Nomograms to predict the 1-, 3-, and 5-year overall survival of patients with high-grade cancers (**a**) and low-grade cancers (**b**). The score for each independent prognostic factor was summed up. Then, the overall survival rate was obtained from the total number of points in the bottom scale for each individual

**Fig. 3:**
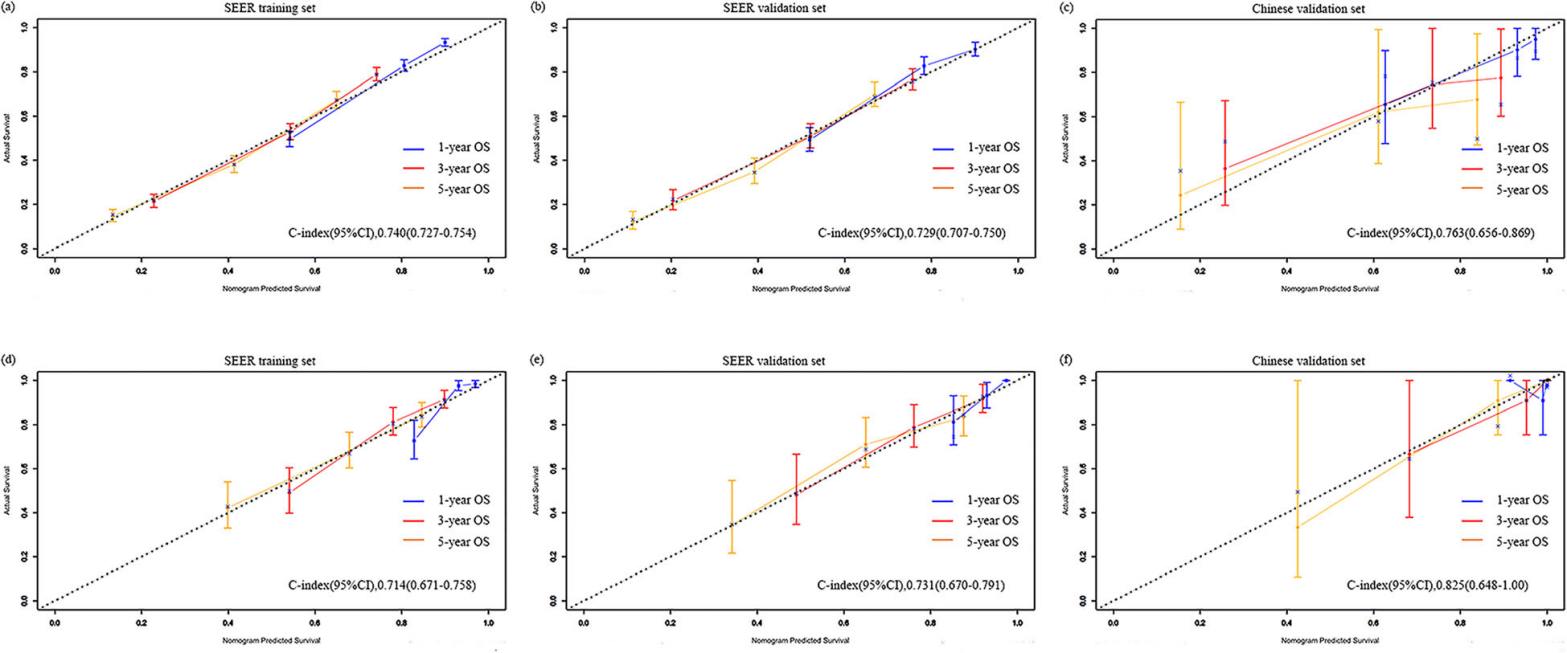
Calibration plots comparing the similarity between the nomogram-predicted survival rates (represented by x-axis) and the actual survival rates (represented by y-axis). **a** 1-, 3-, and 5-year OS for high-grade cancers in the training dataset from the SEER database; **b** 1-, 3-, and 5-year OS for high-grade cancers in the validation dataset from the SEER database; **c** 1-, 3-, and 5-year OS for high-grade cancers in the validation dataset from the China multicenter dataset; **d** 1-, 3-, and 5-year OS for low-grade cancers in the training dataset from the SEER database; **e** 1-, 3-, and 5-year OS for high-grade cancers in the validation dataset from the SEER database. **f** 1-, 3-, 5-year OS for low-grade cancers in the validation dataset from the China multicenter database. OS: overall survival; SEER: the Surveillance Epidemiology, and End Results database

Table 2 shows the results of univariate and multivariate COX regression analysis in the high- and low-grade training dataset. In the high-grade multivariate COX regression results, age at diagnosis, AJCC stage, AJCC TNM stage, tumor size, Lymph node dissection, histology type, radiotherapy, and chemotherapy were significantly related to OS. To get an optimal model, we created two models and compared them. In both models, age at diagnosis, tumor size, Lymph node dissection, pathological type, radiotherapy, and chemotherapy were included. Model 1 included T, N, M stage, and model 2 included AJCC stage. Table 3 lists the C-index of various models; compared to model 2, model 1 showed superior resolution in predicting OS (C-index index, 0.740; 95% confidence interval [CI], 0.727–0.754; *P* < 0.001) (Table 3). Therefore, model 1 was selected as the final model and following these regression results, we drew the corresponding nomogram (Fig. 2a). In addition, the nomogram of the training set showed good calibration (Fig. 3a).

**Table 2:** Univariate and multivariate Cox regression analyses of clinicopathologic factors with overall survival in high- and low-grade SEER training set

Characteristics	Univariable analyses			Model 1			Model 2			Characteristics	Univariable analyses			Model 3			Model 4		
				Multivariable analyses			Multivariable analyses							Multivariable analyses			Multivariable analyses		
	HR	95%CI	P	HR	95%CI	P	HR	95%CI	P		HR	95%CI	P	HR	95%CI	P	HR	95%CI	P
**Age**			**< 0.001**			**< 0.001**			**< 0.001**										
< =66	Ref			Ref			Ref			<=71	Ref			Ref			Ref		
67–80	1.609	1.411–1.833	**< 0.001**	1.585	1.387–1.811	**< 0.001**	1.631	1.427–1.864	**< 0.001**	> 71	2.734	2.008–3.724	**< 0.001**	2.842	2.064–3.913	**< 0.001**	2.938	2.129–4.053	**< 0.001**
> 80	2.567	2.223–2.964	**< 0.001**	2.389	2.051–2.782	**< 0.001**	2.444	2.099–2.846	**< 0.001**		–	–	–	–	–	–	–	–	–
**Sex**																			
male	Ref										Ref			–			–		
female	1.045	0.940–1.160	0.415	–	–	–	–	–	–		1.084	0.800–1.468	0.602	–	–	–	–	–	–
**Race**			0.641			–			–				0.946			–			–
white	Ref										Ref								
black	1.123	0.880–1.434	0.351	–	–	–	–	–	–		1.071	0.502–2.286	0.86	–	–	–	–	–	–
other	1.018	0.856–1.211	0.837	–	–	–	–	–	–		0.915	0.483–1.735	0.786	–	–	–	–	–	–
**Primary_site**																			
Renal pelvis	Ref										Ref			–			–		
Ureter	0.975	0.871–1.092	0.668	–	–	–	–	–	–		1.139	0.831–1.561	0.419	–	–	–	–	–	–
**Laterality**																			
left	Ref										Ref			–			–		
right	1.035	0.932–1.149	0.521	–	–	–	–	–	–		0.954	0.706–1.290	0.761	–	–	–	–	–	–
**Grade**																			
lll	Ref									I	Ref			–			–		
IV	1.006	0.901–1.122	0.916	–	–	–	–	–	–	II	1.313	0.901–1.912	0.156	–	–	–	–	–	–
**Tumor_size**																			
< 65 mm	Ref			Ref			Ref			<=20 mm	Ref			–			–		
> =65 mm	2.085	1.847–2.354	**< 0.001**	1.311	1.141–1.506	**< 0.001**	1.447	1.270–1.648	**< 0.001**	> 20 mm	1.104	0.740–1.647	0.627	–	–	–	–	–	–
**Histology**			**< 0.001**			**0.001**			**< 0.001**										
Transitional carcinoma	Ref			Ref			Ref				Ref			–			–		
Papillary transitional cell carcinoma	0.541	0.486–0.604	**< 0.001**	0.802	0.714–0.900	**< 0.001**	0.779	0.695–0.874	**< 0.001**		0.791	0.566–1.105	0.17	–	–	–	–	–	–
0ther	1.246	0.808–1.921	0.32	1.072	0.694–1.658	0.753	1.194	0.774–1.843	0.422	–	–	–	–	–	–	–	–	–	–
**AJCC stage**			**< 0.001**			–			**< 0.001**				**< 0.001**			–			**< 0.001**
I	Ref						Ref				Ref			Ref			Ref		
II	1.428	1.156–1.764	**0.001**	–	–	–	1.296	1.048–1.604	**0.017**		1.4	0.910–2.153	0.126	–	–	–	1.417	0.920–2.182	0.114
lll	2.139	1.797–2.547	**< 0.001**	–	–	–	1.994	1.667–2.385	**< 0.001**		2.035	1.388–2.983	**< 0.001**	–	–	–	2.153	1.465–3.162	**< 0.001**
IV	5.245	4.409–6.240	**< 0.001**	–	–	–	4.873	3.988–5.956	**< 0.001**		5.699	3.657–8.881	**< 0.001**	–	–	–	4.217	2.499–7.116	**< 0.001**
**T**			**< 0.001**			**< 0.001**			–				**< 0.001**			**< 0.001**			–
T1	Ref			Ref							Ref			Ref			Ref		
T2	1.452	1.190–1.771	**< 0.001**	1.305	1.068–1.594	**0.009**	–	–	–		1.482	0.980–2.240	0.062	1.476	0.973–2.241	0.067	–	–	–
T3	2.384	2.030–2.801	**< 0.001**	2.031	1.716–2.404	**< 0.001**	–	–	–		2.181	1.512–3.147	**< 0.001**	2.223	1.531–3.227	**< 0.001**	–	–	–
T4	5.838	4.851–7.026	**< 0.001**	3.18	2.567–3.939	**< 0.001**	–	–	–		3.932	2.291–6.749	**< 0.001**	2.6	1.383–4.888	**0.003**	–	–	–
**N**			**< 0.001**			**< 0.001**			–				**< 0.001**			**< 0.001**			–
N0	Ref										Ref			Ref			Ref		
N1	2.634	2.268–3.059	**< 0.001**	1.906	1.584–2.294	**< 0.001**	–	–	–		12.262	6.318–23.797	**< 0.001**	5.275	2.238–12.434	**< 0.001**	–	–	–
N2	2.913	2.464–3.444	**< 0.001**	2.284	1.854–2.812	**< 0.001**	–	–	–		23.111	7.079–75.447	**< 0.001**	7.685	1.788–33.028	**0.006**	–	–	–
**M**																			
M0	Ref			Ref							Ref			Ref			Ref		
M1	5.531	4.689–6.525	**< 0.001**	2.889	2.406–3.470	**< 0.001**	–	–	–		11.86	5.750–24.461	**< 0.001**	3.633	1.414–9.333	**0.007**	–	–	–
**Ln_dissection** dissection																			
yes	Ref			Ref			Ref				Ref								
no	0.743	0.667–0.827	**< 0.001**	1.223	1.067–1.401	**0.004**	1.179	1.045–1.331	**0.008**		0.774	0.531–1.127	0.181	–	–	–	–	–	–
**Radiation**																			
yes	Ref			Ref			Ref				Ref			Ref			Ref		
no	0.475	0.398–0.566	**< 0.001**	0.699	0.583–0.838	**< 0.001**	0.732	0.611–0.878	**0.001**		0.268	0.119–0.608	**0.002**	0.814	0.282–2.347	0.703	0.634	0.249–1.613	0.339
**Chemotherapy**																			
yes	Ref			Ref			Ref				Ref			Ref			Ref		
no	0.845	0.751–0.950	**0.005**	1.324	1.157–1.516	**< 0.001**	1.243	1.088–1.420	**0.001**		0.468	0.301–0.729	**0.001**	0.852	0.467–1.555	0.603	0.665	0.388–1.139	0.137

*AJCC* the American Joint Committee on Cancer, *CI* confidence interval, *HR* Hazard Ratio, *Ln_dissection* Lymph node dissection, *SEER* the Surveillance Epidemiology, and End Results database

**Table 3:** Performance of models in the SEER training set

Models	C-index(95% CI)	P*
High grade		
Model 1	0.740 (0.727–0.754)	–
Model 2	0.734 (0.720–0.747)	**0.0006**
Low grade		
Model 3	0.717 (0.673–0.761)	–
Model 4	0.714 (0.671–0.758)	0.474

*SEER* the Surveillance Epidemiology, and End Results database, *AJCC* the American Joint Committee on Cancer

*P values were obtained by comparing model 1 with model 2,as well as model 3 with model 4

**Fig. 4:**
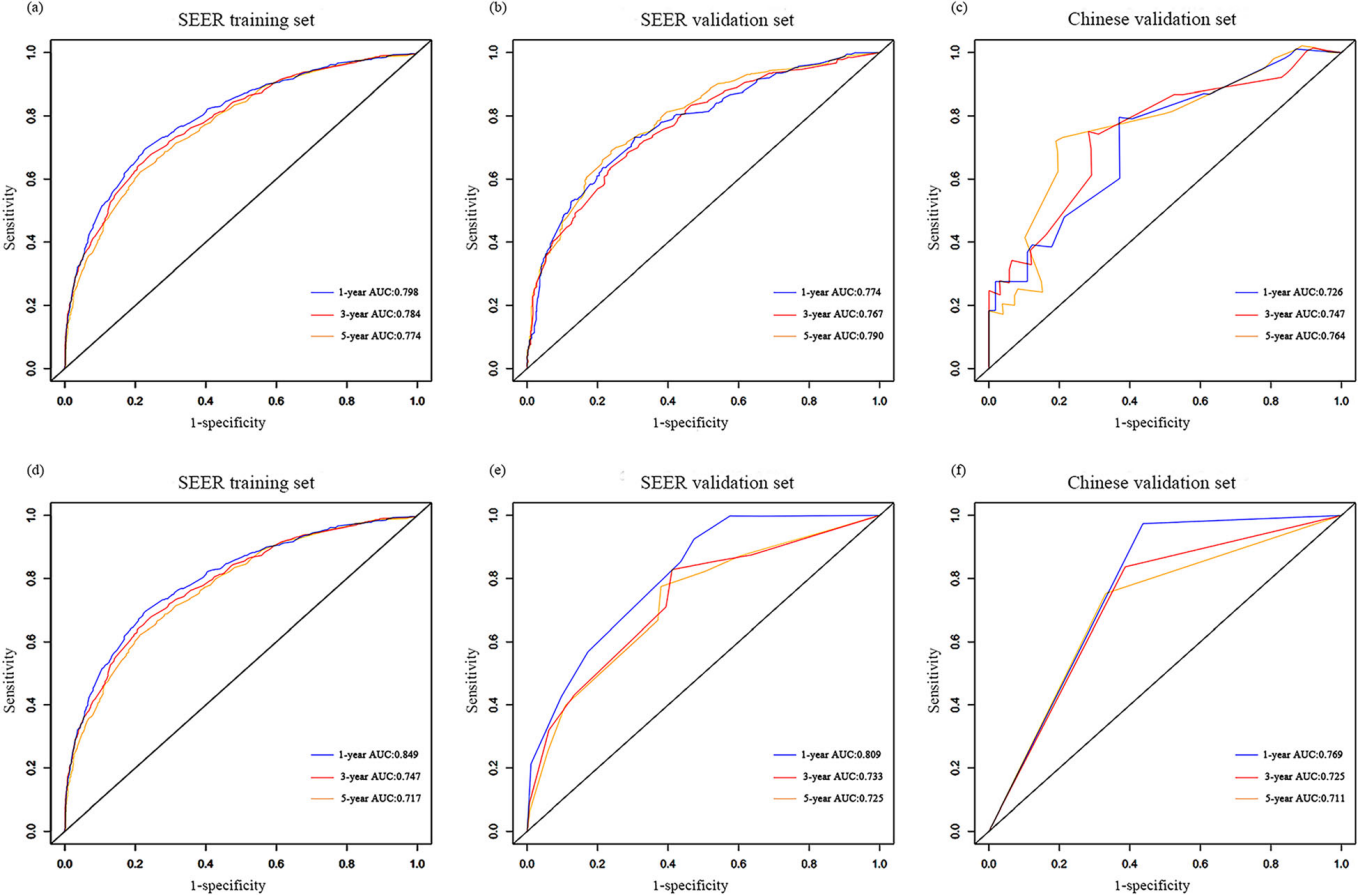
Comparison of the AUC values of the1-, 3-, and 5-year OS for high-grade and low-grade cancers. The AUC values of the nomograms in the training and validation datasets for OS of high-grade (**a**, **b**) and low-grade (**d**, **e**), and the Chinese validation dataset for high-grade (**c**) and low-grade (**f**). AUC: area under the curve; OS: overall survival

The low grade, multivariate COX regression model showed that age at diagnosis, AJCC stage, and AJCC TNM stage were significantly related to OS. Similar to the high-grade, two models (model 3 including T, N, and M stages; and model 4 including the AJCC stage) were established for optimal selection, and age at diagnosis was included in both models. Table 3 shows the C-index results of each model. There was no significant difference between model 3 and model 4 in predicting OS resolution (*P* = 0.474). To ensure a balanced proportion of patients in each stage, model 4 (C index, 0.714; 95% CI, 0.671–0.758) was selected as the best model and a nomogram was drawn (Fig. 2b). The calibration curve of OS within 1-, 3- and 5-year also showed a good calibration of the nomogram (Fig. 3d). The area under the curve (AUC) values of 1-, 3- and 5-year predicted by the nomogram were all greater than 0.7 in the training set for high- and low-grade UTUCs (Fig. 4a and d). Appendix Table 1 shows the regression coefficients.

### Validation of the nomogram

The SEER internal validation dataset (Fig. 3b): C-index,0.729;95\% CI,0.707–0.750) and the Chinese multicenter external validation dataset (Fig. 3c): C-index,0.763; 95\% CI, 0.656–0.869) showed a good recognition ability of the high-grade nomogram. In addition, In the low-grade cohort, both the SEER internal validation data set and the Chinese multi-center external validation dataset showed excellent discrimination on the nomogram, with a C-index of 0.731 and 0.825, respectively (Fig. 3e and f). These results showed that there was good consistency between the validation and training dataset regarding prediction of the nomogram. Therefore, we concluded that the proposed nomogram performed well on both the training set and the verification set. In addition, the 1-, 3- and 5-year predicted AUC values of the SEER internal validation dataset and external validation dataset from Chinese multicenter database were also greater than 0.7 (Fig. 4b, c, e and f).

### Risk stratification based on the nomogram

**Fig. 5:**
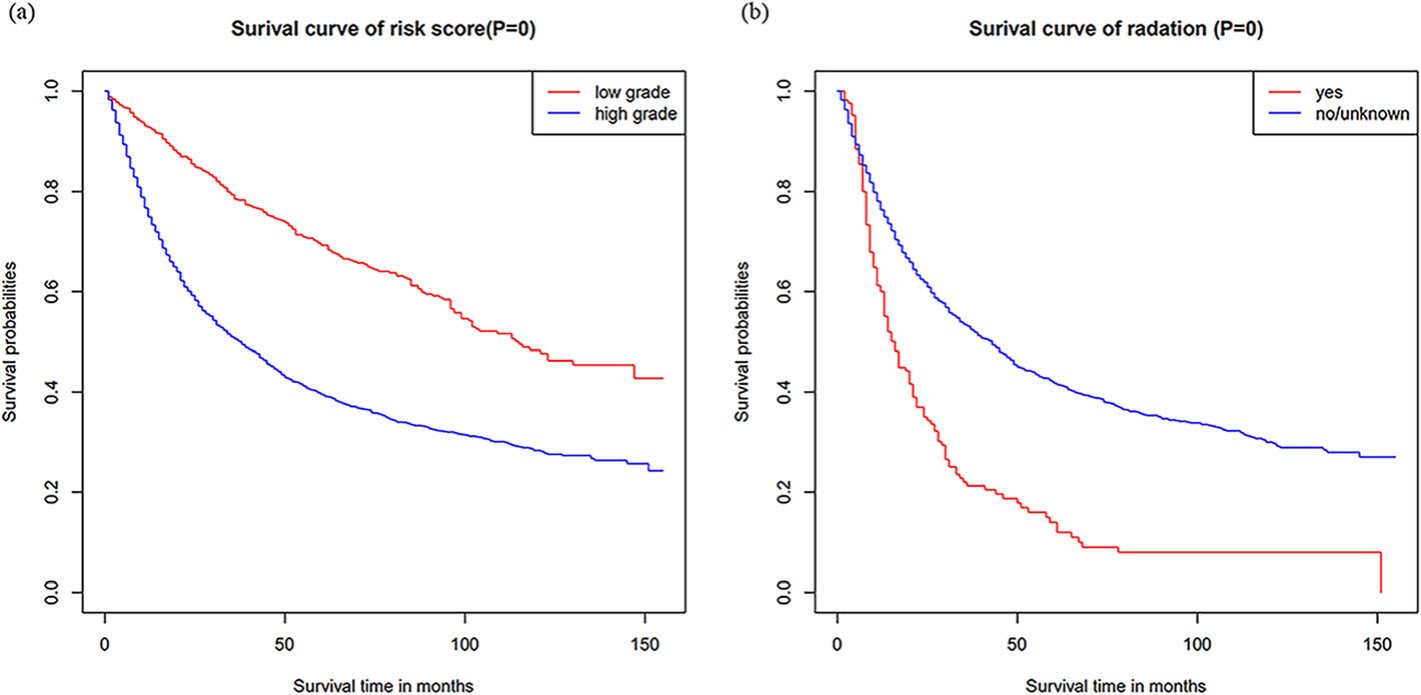
Kaplan–Meier survival curves of patients. Kaplan–Meier survival curves of OS in: (**a**) high-and low-grade datasets (**b**) patients undergoing radiotherapy; OS: overall survival; SEER: the Surveillance Epidemiology, and End Results database

**Fig. 6:**
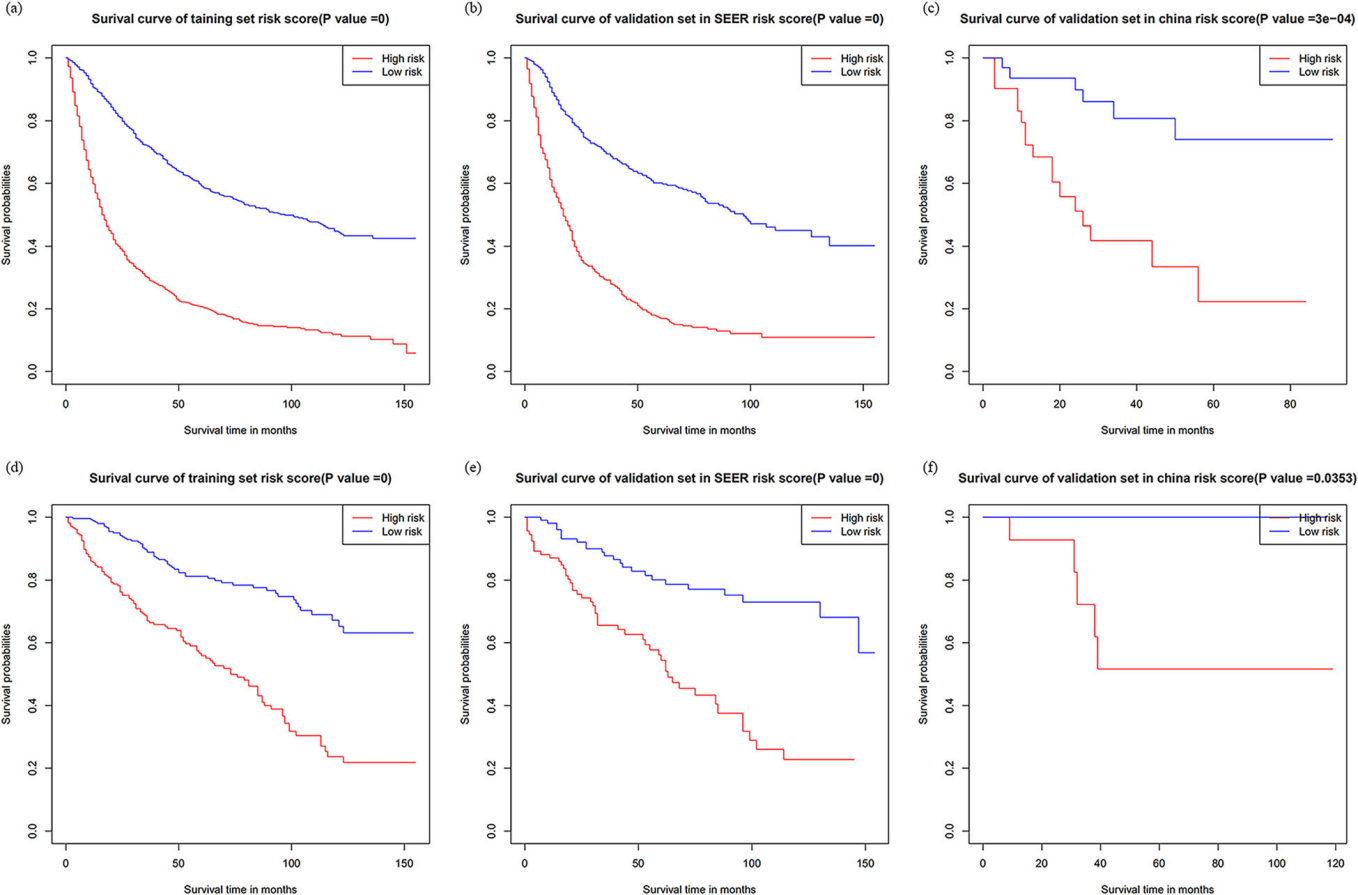
Kaplan–Meier survival curves categorized into low-risk and high-risk groups. **a** high-grade training dataset; **b** high-grade validation dataset in the SEER database; **c** high-grade validation dataset in the China multicenter database; **d** low-grade training dataset; **e** low-grade validation dataset in the SEER database; **f** low-grade validation set in the Chinese multicenter database. OS: overall survival; SEER: the Surveillance Epidemiology, and End Results database

Initially, risk stratification was done for patients with high-and low-grade tumors. The Kaplan-Meier curve showed that there was a significant difference in the survival rate between the two groups, with the low-grade OS being significantly higher than the high-grade OS (Fig. 5a): *P* < 0.001). Finally, according to the total score calculated by the nomogram, the high-grade and low-grade patients were stratified into two risk groups: low-risk and high-risk. In the high-grade training and internal validation datasets and the Chinese multicenter external validation dataset, the Kaplan-Meier OS curves showed significant differences between the two risk groups (Fig. 6a, b and c): *P* < 0.001). The same results were observed for the low-grade datasets (Fig. 6d, e): *P* < 0.001,6(f): *p* < 0.03). In addition, survival analysis of patients on radiotherapy showed significant improvement in OS in patients who did not receive radiotherapy (Fig. 5b): *P* < 0.001). Appendix Figure 2 showed Kaplan–Meier survival curves of patients with UTUC after various surgical procedures.

## Discussion

UTUC is a rare disease. There is currently little clinical understanding of the prognosis of patients with high- and low-grade UTUCs. We conducted survival analysis of patients with low- and high- grades, and the results showed significant differences (*P* < 0.001). Based on the SEER database, this study constructed corresponding prediction models for patients with high- and low-grade UTUC following tumor resection. In the high-grade UTUC patients, nine important factors related to survival after tumor resection were identified, whereas in the low-grade UTUC patients, two important factors related to survival were identified. In addition, the SEER internal verification dataset and Chinese multicenter external verification dataset were used for validation. The results showed that these models exhibited good identification and calibration capabilities. Finally, we used these models to evaluate the risk of patients.

A recent NMIBC EAU Guidelines-panelstudy by van Rhijn et al. support the assumption of reporting both WHO grading scores. In this prognostic factor study the authors found that both classification systems were prognostic for progression but not for recurrence [17].UTUC is naturally similar to bladder cancer, and most studies have been based on the use of 1973WHO classifications, which is a triplex classification system. However, the guideline shows that high and low level dichotomy is an important Risk stratification. In clinical application, the prediction model of classification (2004/2016) also has great clinical significance, but few people have studied its clinical applicability, so it is necessary to discuss and study the high and low levels respectively.

In this study, we found that the 5-year OS after tumor resection was 70.23% for low-grade patients and 40.45% for high-grade patients. Robert et al. found that the 5-year OS for low-grade tumors was 69.4% and that for high-grade tumors was 24.2% [18]. Compared to our study, the OS rate of low-grade patients was similar, but the survival rate of high-grade patients was greatly improved, indicating that although diagnostic techniques and treatment methods have been developed over the years, there is an urgent need to further study survival and clinical features in patients with low-grade UTUCs to guide better outcomes. However, regardless of how the outcomes and endpoints were selected, most studies analyzed patients with high and low grades as a whole, without analyzing the differences in their clinical characteristics. Recently, some studies reported relapse-free survival and postoperative recurrence in high-grade patients [19, 20], whereas there are few studies on these predictions for low-grade patients.

The 2020 guideline proposed an overall cut-off value of 2 cm for high - and low-risk UTUC tumor size. The overall median age after RNU was 69.7 years [4]. However, most previous studies have analyzed the age at diagnosis and tumor size of high and low grades as a whole, and have not analyzed the two separately. In this study, we found that the median age of high-grade patients was higher than that of lower-grade patients, which is similar to the results of Bjarte et al. This finding may be due to the change in tumor cell potential caused by increasing age and the decrease in host defense mechanisms, which makes malignant tumors more likely to occur [13]. In addition, T3 patients accounted for 48.1% of high-grade patients, whereas most of the low-grade patients were T1 (58.2%). The results of Gordona-Brown et al. also supports our observations [21]. The median tumor size was larger in high-grade patients than in low-grade patients (40 mm vs. 32 mm). All of the above analyses showed that high-grade tumors are more invasive and dangerous than low-grade tumors. Importantly, the regression analysis of high-grade patients showed a significant correlation with better survival in patients who received chemotherapy and Lymph node dissection. These results suggest that high-grade patients should be actively treated to improve survival. Compared with high-grade tumors, histological types, tumor size, and treatment were not significantly associated with survival in low-grade patients. Second, low-grade tumors are less invasive and less likely to have recurrence and metastasis. Therefore, follow-up treatment is not heeded, but there are still low-grade patients with a high degree of advanced malignancy [10]. As a result, effective treatment guidance cannot be given to these patients. In summary, we developed a new nomogram to address these problems in patients with high- and low-grade tumors.

Previous studies have shown that T, N, and M stage, age at diagnosis, histological types, Lymph node dissection, tumor size, and treatment are all independent predictors of OS in patients with postoperative UTUC [9]. This is similar to the results of Cox multivariate regression analyses of the high-grade patients in our study. In the process of constructing the high-grade nomogram, TNM stage was the main part of the final risk score compared to the AJCC stage. When combined with other variables, TNM staging showed significant favorable prognostic performance. Therefore, the use of AJCC TNM staging may better guide clinical decisions when predicting prognosis in high-grade patients. For low-grade patients, multivariate Cox regression analysis showed that age at diagnosis was significantly related to AJCC stage and AJCC TNM stage, but the combined AJCC stage did not show a significant correlation with TNM when constructing the model. However, as a single variable, AJCC stage is sufficient and convenient than TNM in overall data collection, so we chose age and AJCC stage as independent predictors for patients with low-grade UTUC. It is important to note that the prediction model has been verified by internal and external data, showing a good prediction efficiency.

The Kaplan-Meier curve showed that in the high-grade dataset, the OS of higher-risk patients was lower than that of lower-risk patients (55.84% vs. 17.53%) (*P* < 0.001). This may suggest that the patient is at a more advanced stage; thus, higher vigilance and active treatment is required when managing these high-risk patients to improve the overall survival rate. In the low-grade dataset, the OS of low-risk patients (81.22%) was higher than that of high-risk patients (57.43%). Therefore, it is necessary to detect and treat such high-risk patients as early as possible in clinical practice to prevent the continued development of their tumors.

Some controversial factors related to the prognosis of UTUC have been found in previous studies. Other studies that have examined adjuvant radiotherapy alone have not shown any benefit on overall survival [9]. Other studies have shown that radiotherapy is associated with a better prognosis [22]. In this study, the Kaplan-Meier curve of high-grade patients receiving radiotherapy showed that the OS of patients receiving radiotherapy was lower than that of patients without radiotherapy. However, the inclusion of adjuvant radiotherapy increased the C-index of the nomogram, indicating its value in predicting OS in patients with UTUC. In addition, more patients with T3/T4 cancers received radiotherapy (88.9%), but the overall survival was worse than that of patients with T1/T2 cancers. Previous studies have shown that patients with T3/T4 cancer who received radiotherapy had a higher OS than those who did not receive radiotherapy. However, regardless of radiotherapy, patients with T3/T4 cancers had a lower OS than patients with T1/T2 cancer [22]. This supports our findings. Second, the majority of patients with radiotherapy are in advanced stages, and the adverse effects of radiotherapy on their survival cannot be ignored. Our nomogram also showed that radiotherapy is an important harmful factor, emphasizing the need to be cautious with patients who are administered UTUC radiotherapy.

In this study, the proportion of various surgical methods of high and low UTUC is shown. The RNU surgical method accounted for the majority of high- and low-grade patients, 57.6 and 60.3%, respectively. Local tumor excision accounted for the least, with 1.2 and 4.1% respectively. Then constructed a survival analysis based on high and low levels. Survival analysis showed that among high-grade UTUC patients, the survival advantage of Partial or subtotal nephrectomy and RNU was significantly better than that of patients with other surgical methods (*p* < 0.001). Among low-grade UTUC patients, the survival advantage of RNU was significantly better than that of patients with other surgical methods (*p* < 0.001). In summary, the RNU operation method has gained more survival time for the patient. In addition to the benefits of the operation itself, it is not ruled out that the postoperative combination of cisplatin chemotherapy can bring more significant advantages to the patient [2].

The variables involved in this study are all clinically easy to collect, and the external verification data show that they are of high clinical applicability. The total number of points calculated from the established nomogram can become a new factor in predicting survival for high and low grade patients. The exciting development is that we achieved excellent discrimination and calibration of the nomogram in the validation dataset. More importantly, the C-index of the China Shandong cohort was 0.763 for the high-grade cohort, and 0.825 for the low-grade cohort. The AUC values of external dataset for high- and low-grade predictive 1-year OS were 0.726 and 0.769; 3-year OS were 0.747 and 0.725; and 5-year OS were 0.764 and 0.711, respectively. In addition, the calibration further confirmed the accuracy of the nomogram between the predicted OS and the observed OS for high- and low-grade patients in Shandong Province, indicating that the nomogram based on the SEER database is also suitable for patients in Shandong Province, China.

Some limitations of this study are as follows. First, this study may be limited due to the retrospective nature of the study. We excluded patients whose data were missing during the data collection process so as not to compromise the credibility of the results. Second, the nomogram may lack some potential predictors because the information was not consistent in the retrieved data set. Such as CKD, tumor multifocality, lymphovascular invasion, specific adjuvant chemotherapy,specific chemoradiotherapy information,template-based manneretc.because these informations were not inaccessible in the SEER database or uniformly available in the Chinese multicenter datasets. A comprehensive nomogram that takes into account all potential predictors may have better prognostic performance. Third, biopsy grade may still be affected by non-negligible sampling error rate [23], especially for large tumors. In order to avoid this effect, the study included patients after surgical resection of the tumor, which may not be applicable for patients with only preoperative ureteral biopsy grading and patients with unknown grade. Finally, even if rigorous tests show very reliable line chart results, the limited range of information obtained from standard clinicopathological variables, the lack of external data, and the short follow-up time lead to insufficient low-grade 5-year follow-up data and imperfect prognostic accuracy. We hope that through continuous research, this model can be extended with additional external data to further improve the prediction of tumor prognosis and optimize patient care.

## Conclusion

In summary, the constructed nomogram provides an easy-to-use tool to guide personalized clinical decisions about management choices when targeting high-and low-grade patients.

## Supplementary Information

**Additional file 1: Appendix Table 1.** Cox regression coefficients of the two models of the SEER training set. AJCC: the American Joint Committee on Cancer; CI: confidence interval; HR: Hazard Ratio; Ln_surg: Lymph node dissection; SEER: the Surveillance Epidemiology, and End Results database.

**Additional file 2: Appendix Table 2.** A table showing the percentage of surgical procedures. Partial nephrectomy: Partial or subtotal nephrectomy (kidney or renal pelvis) or partial ureterectomy; RNU:Complete/total/simple nephrectomy - for kidney parenchyma Nephroureterectomy; Any nephrectomy: Any nephrectomy (simple, subtotal, complete, partial, total, radical) PLUS an en bloc:resection of other organ(s) (colon, bladder); Nephrectomy, NOS:Nephrectomy, NOS;Ureterectomy, NOS.

**Additional file 3: Appendix Figure 1.** (a-d) X-tile plots of age at diagnosis, identifying the best risk score cut-off based on the overall survival (OS) in the high- and low-grades; (e-h)X-tile plots of tumor size, identifying the best risk score cut-off based on the OS in the high- and low-grades.

**Additional file 4: Appendix Figure 2.** Kaplan–Meier survival curves of patients with UTUC after various surgical procedures. (a) Kaplan–Meier survival curves of patients with high-grade UTUC after various surgical procedures (b) Kaplan–Meier survival curves of patients with low-grade UTUC after various surgical procedures. Partial nephrectomy: Partial or subtotal nephrectomy (kidney or renal pelvis) or partial ureterectomy; RNU:Complete/total/simple nephrectomy - for kidney parenchyma Nephroureterectomy; Any nephrectomy: Any nephrectomy (simple, subtotal, complete, partial, total, radical) PLUS an en bloc:resection of other organ(s) (colon, bladder); Nephrectomy, NOS:Nephrectomy, NOS;Ureterectomy, NOS.

## Data Availability

The dataset from SEER database generated and analyzed during the current study are available in the SEER dataset repository (version 8.3.8, downloaded from http://seer.cancer.gov/ seerstat/).
