# Supplementary material for: Construction of a survival prediction model for high-and low -grade UTUC after tumor resection based on “SEER database”: a multicenter study
Source: BMC Cancer. 2021 Sep 7;21:999. doi: 10.1186/s12885-021-08742-3 (PMC8424798; doi:10.1186/s12885-021-08742-3)
Supplement: Supplementary file 2 — Additional file 2: Appendix Table 2. A table showing the percentage of surgical procedures. Partial nephrectomy: Partial or subtotal nephrectomy (kidney or renal pelvis) or partial ureterectomy; RNU:Complete/total/simple nephrectomy - for kidney parenchyma Nephroureterectomy; Any nephrectomy: Any nephrectomy (simple, subtotal, complete, partial, total, radical) PLUS an en bloc:resection of other organ(s) (colon, bladder); Nephrectomy, NOS:Nephrectomy, NOS;Ureterectomy, NOS. [file 12885_2021_8742_MOESM2_ESM.doc]

| **high-grade** | **surgical procedures** | **[cases(%)]** | **low-grade** | **surgical procedures** | **[cases(%)]** |
| --- | --- | --- | --- | --- | --- |
|  | **Local tumor excision**  Local tumor excision, NOS  Electrocautery  Laser ablation  Laser excision  Excisional biopsy | **45(1.2)**  5(0.1)  5(0.1)  8(0.2)  1(0.1)  26(0.7) |  | **Local tumor excision**  Local tumor excision, NOS  Electrocautery  Laser ablation  Laser excision  Excisional biopsy | **28(4.1)**  3(0.4)  6(0.8)  11(1.6)  2(0.2)  6(0.8) |
|  |  |
|  |  |
|  |  |
|  |  |
|  |  |
|  | **Partial nephrectomy** | **319(9.1)** |  | **Partial nephrectomy** | **69(10.2)** |
|  | **RNU** | **2003(57.6)** |  | **RNU** | **406(60.3)** |
|  | **Radical nephrectomy** | **901(25.9)** |  | **Radical nephrectomy** | **128(19.0)** |
|  | **Any nephrectomy** | **135(3.8)** |  | **Any nephrectomy** | **22(3.2)** |
|  | **Nephrectomy, NOS** | **73(2.1)** |  | **Nephrectomy, NOS** | **20(2.9)** |
