# Supplementary figures and images for: Construction of a survival prediction model for high-and low -grade UTUC after tumor resection based on “SEER database”: a multicenter study
Source: BMC Cancer. 2021 Sep 7;21:999. doi: 10.1186/s12885-021-08742-3 (PMC8424798; doi:10.1186/s12885-021-08742-3)

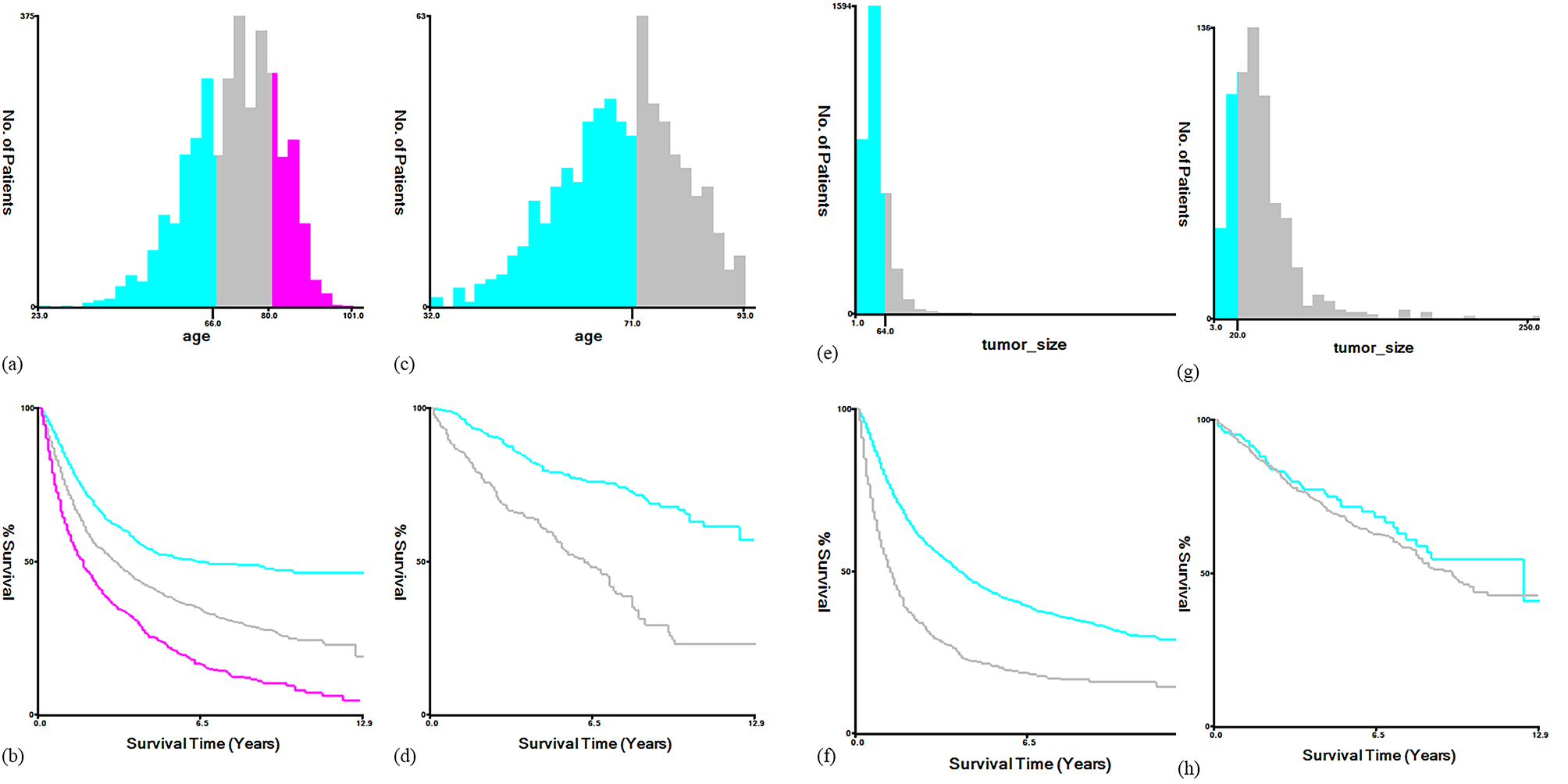

Supplement: Supplementary file 3 — Additional file 3: Appendix Figure 1. (a-d) X-tile plots of age at diagnosis, identifying the best risk score cut-off based on the overall survival (OS) in the high- and low-grades; (e-h)X-tile plots of tumor size, identifying the best risk score cut-off based on the OS in the high- and low-grades. [file 12885_2021_8742_MOESM3_ESM.tif]

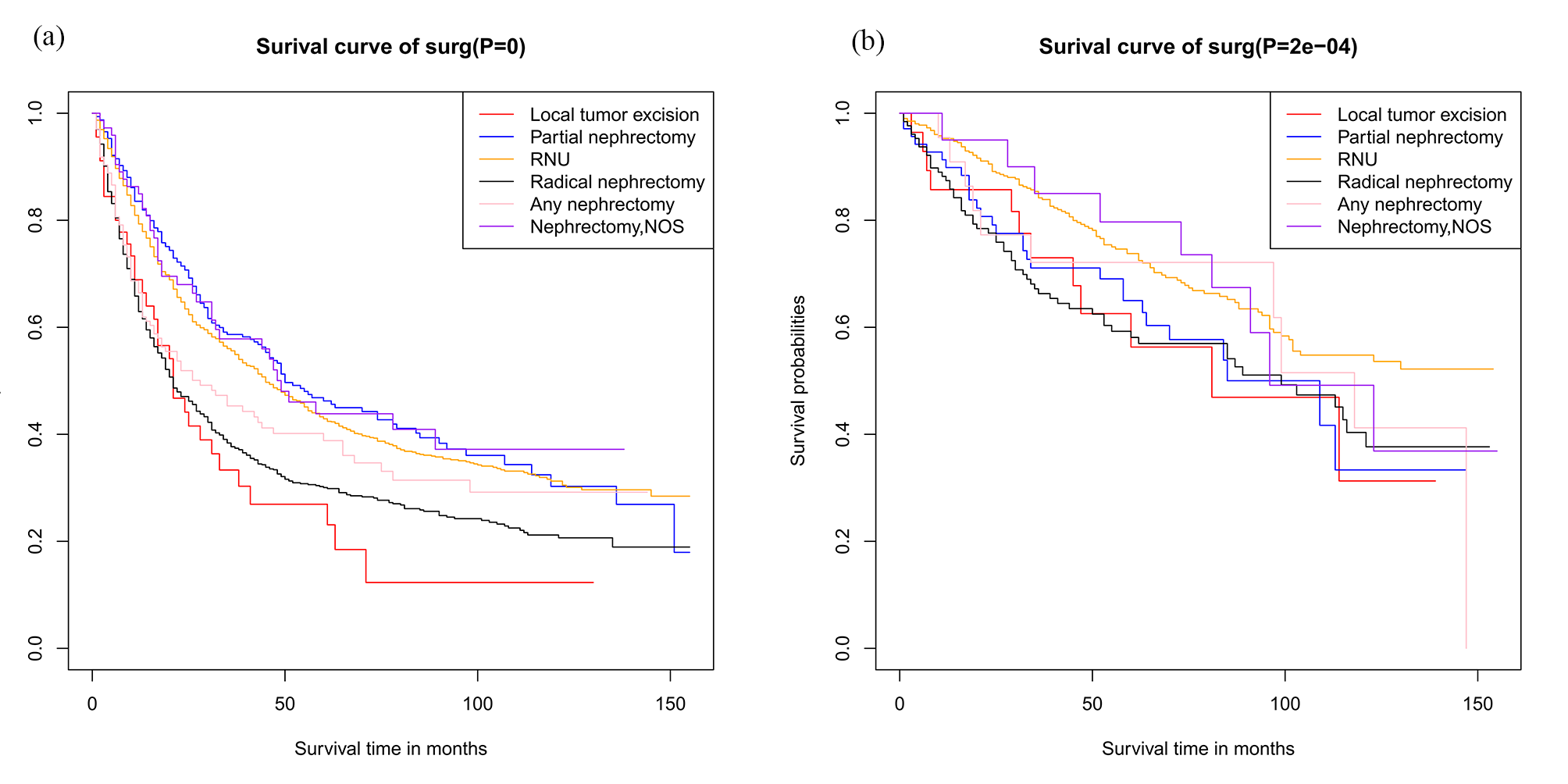

Supplement: Supplementary file 4 — Additional file 4: Appendix Figure 2. Kaplan–Meier survival curves of patients with UTUC after various surgical procedures. (a) Kaplan–Meier survival curves of patients with high-grade UTUC after various surgical procedures (b) Kaplan–Meier survival curves of patients with low-grade UTUC after various surgical procedures. Partial nephrectomy: Partial or subtotal nephrectomy (kidney or renal pelvis) or partial ureterectomy; RNU:Complete/total/simple nephrectomy - for kidney parenchyma Nephroureterectomy; Any nephrectomy: Any nephrectomy (simple, subtotal, complete, partial, total, radical) PLUS an en bloc:resection of other organ(s) (colon, bladder); Nephrectomy, NOS:Nephrectomy, NOS;Ureterectomy, NOS. [file 12885_2021_8742_MOESM4_ESM.tif]
